# P2Y_12_ Inhibitor Monotherapy in Coronary Artery Disease: Translating Evidence Into Practice

**DOI:** 10.31083/RCM44440

**Published:** 2025-09-23

**Authors:** Felice Gragnano, Vincenzo De Sio, Arturo Cesaro, Paolo Calabrò

**Affiliations:** ^1^Department of Translational Medical Sciences, University of Campania “Luigi Vanvitelli”, 81100 Caserta, Italy; ^2^Division of Clinical Cardiology, A.O.R.N. “Sant’Anna e San Sebastiano”, 81100 Caserta, Italy

The optimal antithrombotic regimen following percutaneous coronary intervention (PCI) remains a central issue in the management of patients with coronary artery disease (CAD) [1, 2, 3]. Balancing the ischemic benefits of dual antiplatelet therapy (DAPT) with its inherent bleeding risks is a complex challenge, especially as PCI techniques and stent technologies continue to evolve [4, 5, 6, 7, 8, 9]. Traditionally, DAPT—consisting of aspirin and a P2Y_12_ inhibitor—has been recommended for 6–12 months post-PCI, followed by long-term aspirin monotherapy. However, over the past decade, there has been increased interest in the strategy of early aspirin withdrawal and continuation with P2Y_12_ inhibitor monotherapy, an approach supported by a series of high-quality trials, which have been comprehensively reported in individual patient data (IPD) meta-analyses [5]. This editorial aims to review these pooled analyses, and highlight key messages for clinical practice.

## 1. P2Y_12_ Inhibitor Monotherapy Versus Standard DAPT After PCI

The first landmark meta-analysis published by Valgimigli *et al*. [10] in 2021 on this topic pooled IPD from six randomized trials including 24,096 patients who underwent coronary revascularization, predominately with PCI. The study found that P2Y_12_ inhibitor monotherapy after 1–3 months of DAPT was non-inferior to continued DAPT with respect to major adverse cardiovascular events (MACE), including all-cause death, myocardial infarction (MI), and stroke (hazard ratio [HR] 0.93, 95% CI 0.79–1.09; *p* = 0.005 for non-inferiority). P2Y_12_ monotherapy significantly reduced major bleeding (Bleeding Academic Research Consortium [BARC] 3 or 5 bleeding) by nearly 50%. The results also showed a significant heterogeneity in the treatment effect for sex, indicating that P2Y_12_ inhibitor monotherapy may lower the risk of MACE in females but not in males, driven by a reduction in cardiovascular mortality. The potential benefit on cardiovascular mortality observed in female patients warrants careful consideration and should be interpreted as hypothesis-generating due to the multiple subgroup analyses that were conducted and the absence of correction for multiplicity [10]. Of note, this mortality benefit did not appear to be related to sex-specific differences in bleeding reduction, as both the relative and absolute reductions in major bleeding were similar in males and females. Previously reported sex-related disparities in bleeding management may partly account for this observation, but further investigation is needed [10].

The results of this meta-analysis were further expanded through a secondary analysis stratified by PCI complexity [11]. Among 22,941 patients, 4685 (20.4%) met the criteria for complex PCI. P2Y_12_ monotherapy maintained similar ischemic protection compared with DAPT in both complex and non-complex procedures (*p* for interaction = 0.77), with a consistent and significant 50% reduction in major bleeding across all strata (*p* for interaction = 0.92). These findings challenge the long-standing recommendation that complex PCI mandates prolonged DAPT and instead highlights the potential for simplifying antithrombotic strategies using P2Y_12_ inhibitor monotherapy without compromising efficacy.

A subsequent study advanced the field by evaluating whether the efficacy of P2Y_12_ inhibitor monotherapy depends on the type of P2Y_12_ inhibitor used [12]. In over 25,000 patients, the risks and benefits of ticagrelor monotherapy or clopidogrel monotherapy compared with standard DAPT after PCI were reviewed. Ticagrelor monotherapy was non-inferior to DAPT for MACE (HR 0.89; 95% CI 0.74–1.06; *p* for noninferiority = 0.004) and superior in reducing major bleeding (HR 0.47; 95% CI 0.36–0.62; *p* < 0.001) and net adverse clinical events (NACE) (HR 0.74; 95% CI 0.64–0.86; *p* < 0.001). Clopidogrel monotherapy, however, failed to show non-inferiority for MACE (HR 1.37; 95% CI 1.01–1.87; *p* for noninferiority >0.99), while demonstrating consistent benefit in terms of major bleeding reduction (HR 0.49; 95% CI 0.30–0.81; *p* = 0.006) compared with DAPT, ultimately resulting in a neutral effect on NACE (HR 1.00; 95% CI 0.78–1.28; *p* = 0.99). This evidence suggests that the treatment efficacy of P2Y_12_ inhibitor monotherapy varies depending on the type of P2Y_12_ inhibitor, underscoring the importance of the choice of medication and patient selection when considering clopidogrel as a monotherapy agent.

In 2024, a new meta-analysis focused on the clinical efficacy of ticagrelor monotherapy following short-term DAPT (from 2 weeks to 3 months) compared with standard DAPT after PCI, and whether the treatment effect differs between patients with and without acute coronary syndrome (ACS). In over 24,000 patients, ticagrelor monotherapy demonstrated non-inferior efficacy in terms of MACE (HR 0.91; 95% CI 0.78–1.07; *p* = 0.0039 for non-inferiority) and superior safety for BARC type 3 or 5 bleeding (HR 0.43; 95% CI 0.34–0.54; *p* < 0.0001) compared to 12-month DAPT [13]. The risk of all-cause death was also lower with ticagrelor monotherapy (HR 0.76; 95% CI 0.59–0.98; *p* = 0.034). The benefits of ticagrelor monotherapy were especially evident in ACS patients, and provided data supporting the early discontinuation of aspirin in this high-risk group, which has been traditionally managed with a 12-month course of DAPT.

## 2. P2Y_12_ Inhibitor Monotherapy Versus Aspirin Monotherapy in Patients With CAD

Two additional IPD meta-analyses examined the comparative efficacy and safety of long-term antiplatelet monotherapy with a P2Y_12_ inhibitor versus aspirin. In 2023, the PANTHER meta-analysis compared the two monotherapy strategies using data from seven randomized trials which included 24,325 patients with established CAD, irrespective of their initial treatment (e.g., PCI, coronary artery by-pass grafting, or medical therapy alone) [14]. In the P2Y_12_ inhibitor monotherapy group, 7545 (62.0%) were assigned to clopidogrel and 4633 (38.0%) to ticagrelor. Over two years of follow-up, P2Y_12_ inhibitor monotherapy was associated with a significant reduction in the composite endpoint of cardiovascular death, MI, or stroke (HR 0.88, 95% CI 0.79–0.97; *p* = 0.012) primarily due to a decreased incidence of MIs. Bleeding rates remained comparable between the two antiplatelet strategies. These findings were reinforced by an updated IPD meta-analysis published in 2025 in BMJ [15]. Compared to PANTHER [14], this analysis included newer trials and extended follow-up data from earlier studies, and exclusively focused on PCI patients who had completed DAPT [15]. In 16,117 patients from five trials with a median follow-up of over 3.5 years, P2Y_12_ inhibitor monotherapy significantly reduced the primary efficacy outcome of cardiovascular death, MI, or stroke (HR 0.77; 95% CI 0.67–0.89; *p* < 0.001), without increasing the risk of major bleeding. The number needed to treat for MACE was 45.5, highlighting the clinical relevance of these results.

## 3. Future Perspectives and Conclusions

Collectively, this evidence provides a strong foundation for revisiting the role of aspirin in secondary prevention, particularly in the context of contemporary PCI. Recent clinical guidelines have begun to incorporate these data: the latest American guidelines for the management of ACS endorse ticagrelor monotherapy from 1 month (Class I, A) as an alternative to standard DAPT [3]; the latest European guidelines for the management of chronic coronary syndromes recommend clopidogrel monotherapy (Class I, A) as a safe and effective alternative to aspirin [2]. These updates mark a clear shift toward broader recognition and endorsement of P2Y_12_ inhibitor monotherapy compared with previous guidelines. Although current guidelines do not identify a specific patient subgroup for preferential use of this strategy, available evidence suggests that females may experience increased benefit from P2Y_12_ inhibitor monotherapy. Furthermore, in patients treated with early clopidogrel monotherapy after PCI, genetic or platelet function testing may help to identify poor responders, thereby optimizing antiplatelet efficacy through a more personalized approach.

In summary, P2Y_12_ inhibitor monotherapy has emerged as a viable and often preferable alternative to prolonged DAPT or aspirin monotherapy across a broad spectrum of patients with CAD. This strategy challenges the historical dominance of aspirin in secondary prevention and represents a paradigm shift in antiplatelet therapy, increasingly aligned with contemporary evidence. While current data support early aspirin discontinuation after a short course of DAPT after ACS or PCI, several questions remain. For instance, the perioperative management of patients receiving P2Y_12_ inhibitor monotherapy remains undefined, highlighting the need for standardized protocols to be established in future clinical guidelines.
